# The ASCEND study: protocol for a feasibility study to evaluate an early social communication intervention for young children with Down syndrome

**DOI:** 10.1186/s40814-022-00968-7

**Published:** 2022-01-17

**Authors:** Vesna Stojanovik, Emma Pagnamenta, Emily Seager, Maria Breen, Susie Jennings, Victoria Joffe, Kate Harvey, Elena Pizzo, Hayley Perry

**Affiliations:** 1 School of Psychology and Clinical Language Sciences, University of Reading, Reading, RG6 6AL UK; 2 King’s College London, London, UK; 3 Thames Valley Clinical Trials Unit, University of Reading, Reading, UK; 4 University of Essex, Colchester, UK; 5 University College London, London, UK

**Keywords:** Down syndrome, Intervention, Social communication, Language

## Abstract

**Background:**

Down syndrome is the most common cause of learning disability, affecting approximately 1 in every 700 babies. Children with Down syndrome have particular difficulties with speech and language. This makes it challenging for them to participate fully in life, access healthcare services and educational opportunities. Improving the language skills of young children with Down syndrome is vital for their future social and emotional well-being and behaviour, and consequently contribution to society. As Down syndrome is detected before or at birth, we can provide support from early on. There are currently no standard interventions for improving the language skills of children with Down syndrome under the age of 36 months. Evidence suggests that early parent-based interventions may be effective in improving language outcomes. In partnership with parents and speech and language therapists, we have co-developed an intervention focusing on early social communication skills and our preliminary work shows that it can lead to better language in children with Down syndrome. Our aim is to carry out a feasibility study which will inform a future pilot/full trial to test whether the intervention is effective in improving language skills before children with Down syndrome start school.

**Methods:**

This is a two-arm feasibility randomised controlled trial (RCT), with 1:1 randomisation stratified by trial site comparing the intervention (plus standard NHS speech and language therapy) with no intervention (standard NHS speech and language therapy only). We aim to recruit between 25 and 30 children with Down syndrome aged between 11 and 36 months. Sites are defined by the geographical boundaries of three National Health Service (NHS) Trusts. Recruitment is from NHS Speech and Language Therapist caseloads within the 3 Trusts, and self-referral. In the intervention arm, parents/guardians will receive brief training on the parent-based intervention and a manual to follow with their child for 10 weeks. The children’s language and early communication skills and family health outcomes will be assessed by a blinded assessor at baseline, post-intervention and 6 month follow-up. Questionnaire and semi-structured interviews will explore the acceptability of the intervention to parents and SLTs.

**Discussion:**

The feasibility study’s outcomes will determine whether it would be viable to progress to a full-trial and whether adjustments need to made to the procedures, data collection methods, intervention delivery and the intensity of support needed. We want to assess whether our early intervention can be delivered and rolled out through NHS Speech and Language Therapy (SLT) Services. We anticipate that NHS SLT Services will need to make ongoing changes due to the COVID-19 pandemic, so it is likely that we will need to make adjustments for the definitive trial. We will also calculate descriptive statistics of the language outcome measure which we will use for any future sample size calculation.

**Trial registration:**

ISRCTN13902755. Registered on 25 August 2020. http://www.isrctn.com/ISRCTN13902755

## Background and rationale

### Clinical need

Down syndrome is a genetic condition which affects 1 in 700 live births [1] and is the most common cause of learning disability [2]. There are approximately 38,000 people with Down syndrome living in England and Wales [3]. Language is a particular weakness in this population, and it can even be lower than expected for the level of cognitive functioning. Children with Down syndrome acquire language slowly and often there are severe delays. Some children with Down syndrome enter school with a spoken vocabulary of approximately 300 words [4] compared to typically developing children who have thousands of words [5]. Because language ability at school entry is a predictor of later psycho-social and academic outcomes [6], and early language skills are a primary child well-being indicator [7], it is crucial that children with Down syndrome have as good as possible early language skills, to optimise health and well-being outcomes of both children and their parents. Having a child with Down syndrome can affect parental stress levels and mental health. The stress levels of mothers of children with Down syndrome increases as their child gets older, possibly due to difficulties in accessing services and worrying about their child’s future [8].

Language and communication skills are strongly linked to health outcomes including quality of life, mental health and well-being and access to healthcare. For example, the recommendation to prioritise early language skills as a child well-being indicator was first made by the Equality and Human Rights Commission [9]. Interventions to support language and communication development have been identified by James Lind Alliance Priority Setting Partnership for Childhood Disability [10]. Importantly, the Early Intervention Foundation has recently issued a call to prioritise early language skills as a primary child well-being indicator [7].

Being able to communicate effectively is a critical factor for living safely and independently in the community, and in obtaining the highest quality healthcare [11]. In the twenty-first century, a person’s fitness for survival is determined in terms of their ability to communicate effectively [12]. People with communication disabilities are at risk of not being able to communicate effectively with their healthcare providers, which may directly compromise their health, the healthcare they receive and their right to fully participate in decisions regarding their health [13]. Poor language and communication in childhood has also been linked to poorer long-term outcomes in terms of communication skills, occupational status and educational outcomes [14].

The aim of this project is to empower parents to be able to feel confident in promoting and facilitating their children’s language and communication development from an early age. The Council for Disabled Children emphasises the importance of empowering parents [15]. Improving the language skills of children with Down syndrome in the pre-school years will mean improved quality of life for both children and parents, better health outcomes, reduced parental stress levels and better social and educational participation.

Well-designed interventions focusing on the foundation skills for language are needed to improve language outcomes for children with Down syndrome. Early intervention can be effective, and there is evidence for the effectiveness of early intervention from clinical populations, including children with language delay and autism spectrum disorders [16, 17]. Evidence suggests that the earlier an intervention is delivered, the better the language outcomes [16]. We conducted a systematic review of early interventions for children with Down syndrome under 6 years of age and identified 11 studies involving 242 children, which were either described as RCTs, or included a control group, and had speech/language/communication as primary outcome measure. Although most intervention studies showed positive effects of the interventions on children’s speech and language outcomes, they are hampered by methodological issues and risk of bias [18].

This protocol describes the methods and analysis for a two-arm randomised feasibility trial that will compare the intervention (in addition to standard NHS speech and language therapy) with no intervention (standard NHS speech and language therapy only). The aim is to investigate the acceptability of the intervention and feasibility of a randomised controlled trial and bridge a gap in the current evidence-base for early social communication interventions for children with Down syndrome under the age of 3.

### Early social communication skills as precursors to language

Language development happens in the social context and early social communication skills (before children produce words) are crucial [19–21] and lay the foundation for language to be acquired. A subset of early social communication skills includes, for example, shared attention skills, which is the focus of this research. Shared attention is when the child and parent/caregiver simultaneously focus on the same object or event (for example, both parent and child are looking at a toy simultaneously). If it is the child who chooses an object or topic/event upon which the attention of the child and caregiver is focused, we say that the child has initiated shared attention. When the parent/caregiver chooses a toy/topic to which the infant’s attention is directed, such as for example, the parent points to a toy to get their child’s attention and the child looks at that toy, the child is responding to shared attention. The child’s responding to bids of shared attention results in getting verbal responses from caregivers, which in turn increases the language input the child receives [22] and good quality and quantity of input is crucial in supporting children’s language acquisition [23–26].

Importantly, how well the child responds to the parent/caregiver’s bids for shared attention has been found to be a unique predictor of later language outcomes in children with Down syndrome [20, 27]. Preliminary work by our research team shows that responding to shared attention at 18‑21 months uniquely predicts expressive language outcomes at 32‑35 months in infants with Down syndrome [27]. This finding is based on a longitudinal investigation including 14 children with Down syndrome (aged 18 to 21 months at the start of the study) and 35 typically developing children (matched on non-verbal abilities to the Down syndrome group). All children were seen at 3 time points over 14 months. Interestingly, in typically developing children, speech segmentation skills (and not responding to shared attention) emerged as the longitudinal predictor of language. This difference between children with Down syndrome and the typical children suggests that early social communication skills, and specifically the child’s responding to shared attention, are very important for language development in children with Down syndrome, and an intervention targeting these skills may lead to better language outcomes.

To address this, we have developed an early social communication intervention focused on shared attention. Our preliminary work showed that it can lead to better language in children with Down syndrome [28]. Following this preliminary work, we co-developed a parent-led intervention in collaboration with parents of children with Down syndrome and speech and language therapists designed to be delivered by parents, with the support of brief training and ongoing advice provided by NHS speech and language therapists over 10 weeks.

#### Aims and objectives

The *aim* of this feasibility study is to estimate the parameters to inform a future randomised controlled trial that will evaluate whether the intervention plus standard care is more effective than standard care for enhancing the language and early communication skills and family health outcomes for children with Down syndrome. We want to assess whether our early intervention can be delivered and rolled out through NHS Speech and Language Therapy Services.

The *objectives* are as follows:

- Determine whether parents of children with Down syndrome are willing to be randomised
- Determine the acceptability of the intervention to speech and language therapists and effectiveness of recruitment of children with Down syndrome by speech and language therapists
- Identify different routes to identifying eligible children with Down syndrome (paediatricians, health visitors, speech and language therapists, charities)
- Estimate follow-up rate and adherence to intervention
- Inform the measurement of health economic outcomes and resource implications of a parent-led intervention
- Determine the standard deviation of the primary outcome measure to inform a sample size calculation for a full trial

The effectiveness of the intervention will not be determined in this feasibility study but will be the aim of a subsequent full trial. The feasibility study’s outcomes will determine whether it would be viable to progress to a full-trial and whether adjustments need to made to the procedures, data collection methods, intervention delivery and the intensity of support needed. We anticipate that NHS services will need to make ongoing changes due to the COVID-19 pandemic, so it is likely we will need to make adjustments for the definitive trial.

### Trial design

**Fig. 1:**
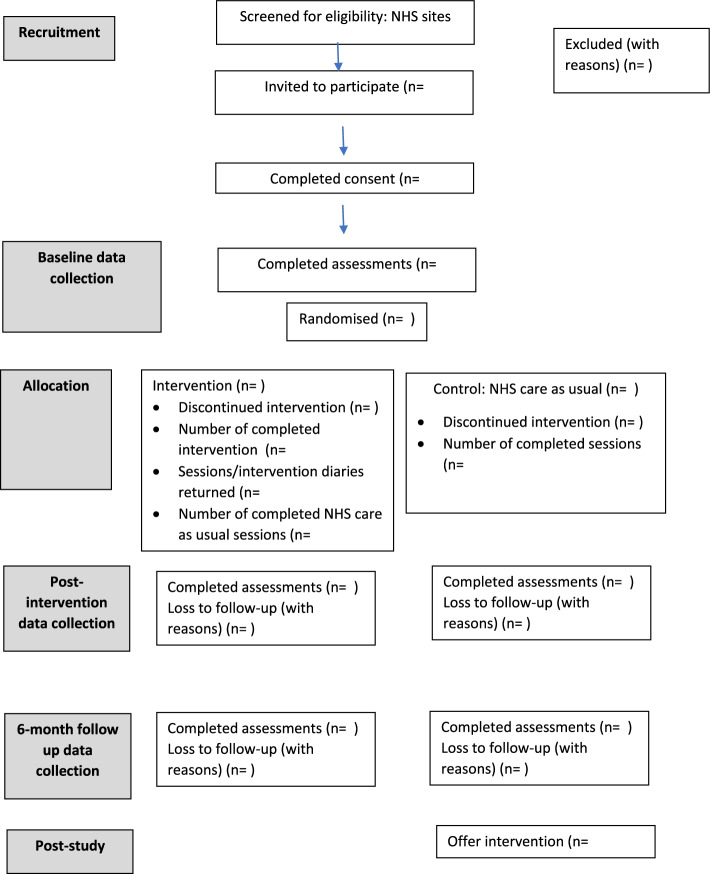
Flow diagram

This study is a two-arm randomised feasibility trial that will compare the intervention (in addition to standard NHS speech and language therapy) with no intervention (standard NHS speech and language therapy only) across three NHS sites. The intervention will be delivered by parents with the support of brief training and ongoing advice provided by NHS speech and language therapists over 10 weeks. The protocol has been developed in line with the Standard Protocol Items: Recommendations for Interventional Trials (SPIRIT) 2013 Checklist. See Fig. 1 for a flowchart of the study.

### Setting

The study will be conducted in 3 NHS sites in England, providing Speech and Language services across three geographical regions: Berkshire Health NHS Foundation Trust (BHFT), Oxford Health NHS Trust (OHFT) and North East London Foundation Trust (NELFT) (http://www.isrctn.com/ISRCTN13902755). All assessments will be conducted remotely using either online or paper questionnaires, with support by telephone. The study is being led by the University of Reading and the lead R&D organisation is BHFT.

### Participants

#### Sample size

Between 25 and 30 children with Down syndrome aged between 11 and 36 months old will be enrolled in this feasibility study over a period of between 6 and 10 months. Given the feasibility nature of the study, no formal sample size calculations were performed; however, the literature recommends a minimum of 24 participants [29–31] in order to derive a standard deviation.

### Eligibility criteria

#### Inclusion criteria

1. Parent or guardian willing and able to provide informed consent on behalf of participant.
2. Confirmed diagnosis of trisomy 21 (Down syndrome).
3. Male or female child, 11 to 36 months old at study entry.
4. Parent/guardian has the literary and language skills needed to use intervention manual.
5. The participant has not previously been entered into this study.
6. The child is not currently taking part or planning to take part in a language-based intervention study.

#### Exclusion criteria

1. Children with co-morbid conditions (for example Down syndrome and Autism spectrum disorder) as determined by the Principle Investigator.
2. Any reason in the opinion of the Principal Investigator that the child is not suitable for study participation.
3. Prior knowledge of the intervention as specified in the parent manual.

### Intervention

The intervention focuses on promoting the development of early social communication skills and in particular, the child’s ability to respond to shared attention. The parent/guardian will be provided with an intervention manual (paper based), a bag of age-appropriate toys and links to short video demonstrations of the intervention, with explanation from either the PI or the child’s speech and language therapist. The intervention will be administered in the child’s home by the parent/guardian. The parent/guardian will be advised to progress through the stages of the intervention step by step and practise 3‑6 times a week, totaling an hour a week, for 10 weeks. Support to deliver the intervention from the child’s speech and language therapist (SLT) (all of whom have had training on how the intervention works) will be available at the request of parents by telephone/email. Parents also have access to the PI of each site. The SLT will record all contacts from parents in the child’s case notes including duration and content of each contact. There will be two telephone calls from the PI, one at week 4 and the second at week 8 to offer support and check and ensure adherence to the intervention manual. They will ask a standard set of questions including how closely parents are following the manual, how often they do the intervention and for how long.

The participants in the intervention group will continue to also receive their usual care.

#### Comparator

The comparator is standard NHS speech and language therapy for this patient group.

### Procedure

Speech and language therapists from participating NHS Trusts will identify potential participants (children with Down syndrome) and their parents by reviewing their caseloads against the inclusion criteria. We will also publicise the study via other healthcare professionals and charities to give other potential participants the opportunity to self-refer to take part in the study.

The speech and language therapists (standard care team) will introduce the study to the parents of all potentially eligible children at a routine appointment, or will contact parents specifically to introduce the study, and ask them if they may be interested in taking part. The parent will be provided with a participant information sheet giving details of what study participation will involve and asked to contact the research team if they may be interested in taking part, or if they have questions.

Parents will have as long as they need to consider participation and the opportunity to ask the speech and language therapist, the research team or other health professionals as many questions as they like in order to make their decision. Parents may choose to delay study entry to fit in with other family commitments. Where possible, reasons will be gathered if a family chooses not to participate by the child’s speech and language therapist.

Self-referring participants will receive the same information and have the same opportunities to discuss the study with members of the research team and ask any questions before they decide whether they would like to take part. If they do not currently have an NHS speech and language therapist, they will be advised to make a self-referral to their local service.

If they wish to take part, parents/guardians will contact the research team who will provide an electronic copy of the consent form. The consent form must be completed prior to commencement of any study activities. Given the age of the study population, 11‑36 months old, the children will not be provided with information about the research nor will their assent be sought.

#### Randomisation

Once informed consent has been given and baseline assessments have been completed, participants will be randomised by the clinical trial manager or other designated team member via Sortition® (a secure web-based clinical trial randomisation software developed by the University of Oxford) using block randomisation to receive standard care (Control) or standard care plus the intervention (Intervention) in a 1:1 ratio, stratified by site, to account for regional differences in standard care. Following randomisation, the parents/guardians will be contacted by the study team, who will explain their child’s study allocation and what will happen next.

#### Intervention group

Parents will be provided with a copy of the intervention manual (paper), a standard set of age-appropriate toys and books, a blank parent treatment diary and guidance on how to complete it and a study schedule of planned conversations with members or the study team and when to complete the assessments. The study team will remind parents of the importance to the study findings of not sharing the manual with other parents.

Parents will be asked to use the manual to support their child in developing their skills in responding to bids for shared attention. The parents will be asked to follow the manual stages and undertake 1 h of the intervention every week, split into several short sessions (3‑6 per week), in whichever way the parent chooses. Parents will be asked to record the date and duration of each session, how many toys they used and which stage they are working at, in the diary provided. Parents are asked to return electronically the diary weekly to the clinical trial manager.

Parents who do not complete the diary will be contacted by telephone or email at the end of the intervention period to remind them to return the diary, or to investigate the reasons for non-adherence. Two attempts will be made to contact parents, in order to respect their privacy and right to discontinue participation.

#### Control group

The children allocated to the control group will receive the standard care for this patient group. SLTs will record all contacts with the family for the duration of the study in terms of duration and activity (assessment, advice, intervention).

At the end of the 6-month follow-up period, they will be provided with the intervention manual, accompanying materials and corresponding SLT or member of the study team support.

#### Adherence to the intervention and contamination

Adherence to the intervention will be monitored by using a weekly diary. The Principal Investigators/Site Leads will phone the parents twice during the intervention period: in weeks 4 and 8 to check adherence, with a window of +/− 5 days. They will ask a standard set of questions including how closely parents are following the manual, how often they do the intervention and for how long. Having this information will allow us to have a log that can provide qualitative data as well as act as a metric of whether the intervention was acceptable to the parents and SLTs.

Any contamination that occurs between the intervention group and the control group will be measured at study entry. Parents will be asked whether they are familiar with the intervention materials, whether they have discussed the intervention with other parents or seen the manual and whether they may have carried out any activities as described in the manual. This will be part of a questionnaire that asks about previous interventions the children may have had. During the post-randomisation process, when parents are contacted by a member of the study team who will explain to them what the allocation means, they will be reminded that the materials are for their own use only and with their own child only and are not to be shared with other parents. In addition, the fact that the control group will be given access to the manual and accompanying materials at the end of the study should minimise contamination risks, as parents will be less likely to want to get hold of the manual via other parents.

### Data collection

All children in the intervention and in the control group will be assessed by an assessor blind to group allocation before randomisation/pre-intervention, immediately post intervention (3 months after) and at a 6-month follow-up, using the following instruments:

- Reading Communication Development Inventory [32], a parental checklist which assesses receptive and expressive language. This is also the primary outcome measure.
- Communication and Symbolic Behaviour Scale [33] which is a norm referenced instrument available as an online or paper questionnaire completed by parents/caregivers and assesses communicative functions, gestural communicative means, vocal communicative means, verbal communicative means, reciprocity, social-affective signalling and symbolic behaviour. This is the secondary outcome measure.
- Vineland Adaptive Behaviour Scale [34] assesses general cognitive and adaptive abilities and is completed by parents.
- Infant Toddler Quality of Life (ITQOL-SF47) [35] is a measure of infant quality of life.
- Adult Quality of Life Questionnaire [36]
- Hospital Anxiety and Depression Scale [37]

Participants will also complete a demographic questionnaire at baseline. All questionnaires will be completed by parents online or using paper copies posted to the participant (if that is what the parent wishes), with support from a member of the research team, if required.

### Outcomes

The effectiveness of the intervention will not be determined in this feasibility study. This will be assessed in a subsequent pilot/full trial. The feasibility study’s outcomes will determine whether it would be viable to progress to a full-trial and whether adjustments need to be made to the procedures, data collection methods, intervention delivery, intensity and support needed. We will also calculate descriptive statistics which we will use for any future sample size calculation.

### Objective 1: Are parents of children with Down syndrome willing to be allocated to ‘standard care’ versus ‘standard care plus parent-led intervention’

During their meeting/telephone conversation or through email contact with their speech and language therapist (SLT), parents will be invited to take part in the study. The SLT will explain that if the parents agreed to participate, they would be randomly assigned to either ‘standard care’ (i.e. will get our intervention at a later time point), or standard care plus parent-led intervention group. If parents decline to participate, the SLT will invite them to state a reason (it will be important to know for a future full trial what the reasons for not accepting the intervention may be so these could be addressed), whilst assuring the parents that their decision will not affect their child’s future health provision. Parents’ responses will be coded using thematic analysis.

### Objective 2: Acceptability of the intervention to speech and language therapists and their willingness/effectiveness to assist with participant recruitment

It is important to understand the speech and language therapists’ willingness to recruit to a future randomised control trial and support parents in the delivery of a parent-led intervention. To address this objective, all SLTs who had facilitated recruitment and/or the delivery of the intervention during the feasibility study, as well as other SLTs from Oxfordshire, Berkshire and other counties with paediatric caseloads will be invited to participate in an interview with a member of the research team. From this pool of participants, we will purposively sample so that all SLTs who had supported the delivery of our intervention during the feasibility study (10‑12) take part, and SLTs who were not involved in the feasibility study with a range of specialisms are also represented. Potential participants will be invited to take part in a one-to-one interview (face-to-face or by telephone) with a member of the research team, who will use a topic guide developed in collaboration with the Public and Patient Involvement group. They will be audio-recorded and transcribed verbatim. Data will be collected until theoretical saturation is reached [38] (around 20‑25 interviews). Data will be coded using NVivo (v11) and analysed using Framework Analysis [39].

### Objective 3: Explore different methods to identify eligible children with Down syndrome and other potential sites

Although speech and language therapists are the obvious professionals to help recruit children with Down syndrome, not all children will be receiving support from SLT services and may still benefit from the intervention; hence, we will also explore other routes, including paediatricians and charities which focus on supporting children with Down syndrome. The chief investigator has collaborated with DownsEd International and Breakthrough Learning, and both charities support individuals with Down syndrome. We will also make links with other potential NHS sites (so we have a bigger pool of sites for a future pilot/full randomised control trial).

### Objective 4: Estimate retention and completion rates, adherence and acceptability of intervention to parents

The trial manager will keep a recruitment log to determine participation, adherence, drop-out and completion rates. These data will also be used to determine how long it would take to recruit participants into a full trial and the number of SLTs and sites needed. A parent weekly diary given to the parents will measure treatment intensity, completion rates and adherence to intervention. After completing the intervention, parents will be sent a questionnaire relating to their experience of the intervention, for example ‘how easy was it to follow the manual’.

### Objective 5: To inform the measurement of health economic outcomes and resource implications of a parent-led intervention

We will identify how best to collect information about health outcomes for the children with Down syndrome and their parents/carers as well as resource implications for the health service. Before the feasibility study commenced, we recruited 6 parents of children with Down syndrome to take part in a focus group. The aim was to help us identify the most appropriate health outcome measures they thought would be useful to include in the feasibility study. These parents had older children with Down syndrome and hence their children were not eligible to participate in the current feasibility study. The measures selected by the focus group (Adult Quality of Life Questionnaire, the Hospital Anxiety and Depression Scale, and the Infant Toddler Quality of Life Questionnaire) will be administered pre-intervention, immediately post-intervention and 6 months post-intervention.

### Objective 6: To determine the standard deviation of the outcome measure, which is needed to estimate sample size for a pilot/full trial

To estimate the standard deviation of the outcome measure, the literature recommends a minimum of 24 participants [29–31]. Descriptive statistics of the language measure (the Reading Communicative Development Inventory) will be derived.

### Data analysis and presentation

Given the feasibility objectives of this study, the focus of data analysis will be descriptive. Recruitment and retention rates will be summarised and presented as a consort diagram. The number of participants enrolled, the number and percentage completing and withdrawing along with reasons for withdrawal will be summarised by intervention arm. Adherence to the intervention will also be summarised.

The language outcome measure (the Reading Communicative Development Inventory) will be summarised using descriptive statistics by visit (time-point) and intervention arm. The Reading Communicative Development Inventory will also be analysed using mixed effect model for repeated measures with terms for baseline, site, age, intervention group, visit, baseline by visit and visit by intervention group. Repeated measures on a participant will be accounted for. Adjusted means, treatment differences and associated 95% confidence intervals will be presented.

The amount and percentage of missing data will be presented. Further exploratory analyses may be performed.

### Health economics/health outcomes

The health economics component will explore the feasibility to identify and gather the relevant data required to evaluate the cost-effectiveness of the parent led intervention compared to usual care within a future full RCT. We will identify the intervention and trial costs (cost for manual, SLT costs, cost for material used in the intervention, cost of phone calls) from an NHS and personal social services perspective. We will also identify potential cost offsets and healthcare benefits. A descriptive analysis will be performed to assess the cost of the intervention and changes in health outcomes. During the feasibility study, we will explore if there are more specific instruments that might be used to capture changes in healthcare status of children and their development using the IQToL and their parents/guardians using the AQoL and HADs. We will also investigate the possibility of capturing costs for families (private costs related to time and productivity losses). If the results are good, we could potentially run a within study economic evaluation to compare the changes in costs of the new intervention compared to the old one and the changes in outcomes.

### Data management and security

#### Access to identifiable and sensitive information

Direct access to the study data will only be given to authorised representatives from the sponsor and host institution for monitoring and/or audit purposes to ensure compliance with regulations. Study staff will only have access to identifiable participant data if it is (1) in line with the informed consent given and (2) is essential for them to carry out their study role. Data will be de-identified as soon as it is practical to do so. The processing of the personal data of study participants will be minimised by use of a unique participant study ID on all study documents.

Research data will be kept secure and confidential, and access to personally identifiable data will only be granted to appropriate researchers and clinicians within the study research team. All questionnaire data will be labelled with numeric identifiers and data linking the identifier to patient information will be kept in a restricted access file separate to any other patient data.

#### Data transfer, storage and archiving

All data transfer will be in line with the sponsors standard operating procedures and the informed consent provided. Electronic Case Report Forms (CRF) will be stored securely and password protected on University of Reading computers. The Chief Investigator will act as custodian of the data and ensure all regulatory and legal requirements are adhered to with support from the study sponsor. Personal data will be stored for 5 years after the study has ended.

### Study governance

Project management will be organised at a number of levels, with a part-time trial manager working alongside the chief investigator (CI) and all the principal investigators. The CI has overall responsibility for the trial.

A Trial Steering Committee (TSC) will be established to provide oversight of the study. The TSC will meet 3 times in the first year and twice in the second year of the project. This committee includes an independent chair, independent statistician, a speech and language therapist not involved in the trial, the CI, all PIs, at least one public and patient involvement representative, and a person responsible for governance from the host organisation. The Steering Committee is chaired by an independent person (somebody who is an experienced Chief Investigator but not involved in the project). These meetings cover recruitment, adherence to protocol, monitor the rights and well-being of participants, finance and all NHS related issues. The Trial Steering Committee will monitor the acceptability of the treatment and any potential adverse effects.

The sponsor will be provided with direct access to source data and other documents if required for trial review.

### Dissemination

The results of this study will be reported in peer reviewed scientific journals, conference presentations, publication on a website. The participants will be provided with a lay summary. Access to raw data and right to publish freely by all investigators in study or by independent steering committee on behalf of all investigators.

## Discussion

This study is the first-step in the development of an evidence-based theoretically-driven early social communication intervention for children with Down syndrome, bridging a gap in the current evidence-base for intervention for very young children. This feasibility study will allow us to investigate if it is possible to deliver this kind of intervention in the context of NHS speech and language therapy services and also determine whether a full RCT to test the effectiveness of the intervention is viable.

If rates of recruitment, retention and data completeness are adequate, and the study procedures are reported to be acceptable to SLTs and participants, we will consider conducting a full trial. These are our success criteria and barriers to proposed work:

### Recruitment

#### Success

We recruit at least 25 children with Down syndrome from three sites within a period of 10 months.

#### Barrier

Low recruitment

#### Contingency

We will have a recruitment strategy to approach all relevant services (speech and language therapy, health visitors, occupational health, physiotherapy, peadiatrics, etc.) which will be regularly reviewed to closely monitor recruitment rates and try to find out the reasons why parents may not be willing for their children to be part of the study which we will take into account in a future pilot/full trial.

### Randomisation

#### Success

At least 70% of parents who are interested in being part of the study are willing to be randomised.

#### Barrier

More than 30% of parents are not willing to be randomised.

#### Contingency

We will investigate the reasons for this and make suitable adaptations to our recruitment procedure in a future pilot/full trial.

### Intervention

#### Success

The intervention is acceptable to 80% of the SLTs taking part in interviews about the acceptability of this kind of trial and who have children with Down syndrome on their caseloads, are willing to assist with recruitment and support the families.

#### Barrier

More than 20% of the SLTs do not accept the intervention.

#### Contingency

We will find out the reasons for why the intervention may not be acceptable and work with SLTs on a possible solution.

#### Success

The intervention is delivered according to the protocol.

#### Barrier

Delivery is problematic or below acceptable quality.

#### Contingency

Site leads and the trial manager will monitor adherence, completion rates and potential contamination.

Information gathered from this feasibility study will enable us to make any necessary improvements to trial procedures and to calculate the sample size required for the definitive trial. The health outcomes data will enable evaluation of the cost-effectiveness of rolling out the intervention to NHS services.

### Trial status

This paper refers to protocol version 1.4 dated 15 March 2021. Recruitment began on 9 September 2020 with completion expected by 30 June 2021. Post-intervention assessments are expected to be completed by the end of March 2022.

## Data Availability

Not applicable
